# The role of lifestyle changes in the management of chronic liver disease

**DOI:** 10.1186/1741-7015-9-70

**Published:** 2011-06-06

**Authors:** Valerio Nobili, Christine Carter-Kent, Ariel E Feldstein

**Affiliations:** 1 Metabolic and Autoimmunity Liver Unit, "Bambino Gesù" Children's Hospital and Research Institute, S. Onofrio Square, 4, 00165 Rome, Italy; 2 Department of Pediatric Gastroenterology, Cleveland Clinic, 9500 Euclid Avenue, Cleveland, OH 44195, USA; 3 Department of Cell Biology, Cleveland Clinic, 9500 Euclid Avenue, Cleveland, OH 44195, USA

## Abstract

The prevalence of obesity worldwide has dramatically increased during the last three decades. With obesity comes a variety of adverse health outcomes which are grouped under the umbrella of metabolic syndrome. The liver in particular seems to be significantly impacted by fat deposition in the presence of obesity. In this article we discuss several liver conditions which are directly affected by overweight and obese status, including non-alcoholic fatty liver disease, chronic infection with hepatitis C virus and post-liver transplant status. The deleterious effects of obesity on liver disease and overall health can be significantly impacted by a culture that fosters sustained nutritional improvement and regular physical activity. Here we summarize the current evidence supporting non-pharmacological, lifestyle interventions that lead to weight reduction, improved physical activity and better nutrition as part of the management and treatment of these liver conditions.

## Introduction

Severe liver disease has significant effects on an individual's quality of life (QOL) as well as their life expectancy. The treatment of many of these conditions involves multi-drug regimens which can be associated with a variety of side effects. Although therapy with prescription medication is often unavoidable, maximizing lifestyle interventions can play a key role in maintenance of overall health augmenting medical therapy in patients with chronic liver disease. Recently published studies have advanced our understanding of how various lifestyle interventions may improve the pathology and symptoms related to specific liver diseases, thereby improving quality of life for these patients.

Lifestyle modifications are strongly recommended for patients with non-alcoholic fatty liver disease (NAFLD), hepatitis C virus (HCV), as well as patients who have undergone liver transplantation. Even when efficacious pharmacologic interventions are identified, lifestyle changes will likely represent an adjuvant treatment because new drugs are inevitably expensive and may have unanticipated adverse effects after prolonged use. These lifestyle modifications typically encompass both dietary intervention and physical activity goals. Unfortunately, these straightforward goals are surprisingly hard to achieve in an environment of copious amounts of high-calorie food and busy daily routines that seem to preclude time for exercise. Many of these difficulties can only be overcome by a culture change that values wholesome foods and regular exercise over the convenience of prepared foods and sedentary activities. In the following sections we will discuss the current evidence for non-pharmacological, lifestyle interventions in the management of some of the most common causes of chronic liver disease including NAFLD, chronic HCV infection, as well as in the liver transplant population.

## Non-alcoholic fatty liver disease

It has been well documented in the medical literature that several types of hepatic conditions are dependent upon or worsened by the presence of obesity in patients. It has become clear that obesity and features of the metabolic syndrome, which encompass glucose dysregulation, dyslipidemia and hypertension, are key risk factors for the development and progression of NAFLD [1]. The presentation and severity of NAFLD can vary significantly, from simple steatosis (which represents fatty deposition without inflammation) to non-alcoholic steatohepatitis or NASH (which encompasses steatosis, inflammation and ballooning degeneration of hepatocytes) to liver fibrosis and end-stage liver disease. Due to increasing rates over the last few decades, NAFLD is now considered to be the most common form of chronic liver disease in most of the Western World, with a prevalence ranging from 20 to 35% of adults and 5 to 17% in children [2,3]. NAFLD has a close association with disordered insulin utilization in the body, thus patients with NAFLD often suffer from insulin resistance and Type II diabetes. Multiple studies have been performed detailing the relationship between insulin resistance and NAFLD and have shown that disordered insulin regulation results in multiple, altered metabolic pathways which ultimately lead to fat deposition and inflammation in the liver [4,5]. Patients diagnosed with metabolic syndrome are at high risk for developing cardiovascular disease including myocardial infarction and stroke. Further, a growing body of evidence suggests that the presence of NAFLD is a risk factor for cardiovascular disease independent of the presence of other features of metabolic syndrome [6,7]. Given the fact that these patients are at risk of both severe liver damage and cardiovascular disease, efforts at weight loss and lifestyle changes are paramount in their overall care. In addition, although there is intense research to identify effective medications for this condition, there is currently no consensus for the use of any single drug or drug combination for treatment of NAFLD.

Over the last few years there have been several studies published which have attempted to determine the effects of either diet and/or exercise on NAFLD; however, most of these studies have been limited due to the small number of patients studied, the short duration of intervention, and the lack of control arms. Table 1 summarizes the main features of these studies. Uneo and colleagues were one of the first groups to report on the effects of diet and exercise on NAFLD. They demonstrated that a short-term, restricted diet and exercise program lasting three months led to a decrease in liver enzymes, improved cholesterol and blood glucose levels in patients with NAFLD [8]. In their cohort of patients who completed the diet and exercise program, body mass index (BMI) decreased from 31 kg/m^2 ^to 28 kg/m^2^, while the control group gained weight. More recently, St. George *et al. *examined the impact of a behavior change-based lifestyle intervention on physical activity levels and the effects of these changes on the metabolic profile of patients with fatty liver disease [9] They reported that enhanced physical activity alone favorably impacted patients with NAFLD including reduction in both aminotransferase levels and other metabolic indices. Notably, the effects of these changes were independent of weight loss. Both of these studies imply that lifestyle modification in the short-term (three months duration for each study) is successful in improving not only the effects of NAFLD, but also other features of the metabolic syndrome for these patients. In addition, a 2009 study evaluated the effects of four weeks of an aerobic exercise program on NAFLD and obesity utilizing MRI findings related to NAFLD before and after the exercise intervention. The authors reported a mean reduction of hepatic triglyceride content by 21% as well as a reduction of visceral adipose tissue volume by 12% for patients who participated in the exercise program [10]. Importantly, these changes were in the absence of weight loss, suggesting a key role for fitness in the reversal of hepatic lipid accumulation. Examining the effects of diet alone in patients with NAFLD, Huang *et al*. reported that after one year of intense nutritional therapy, 60% of patients experienced histologic improvement of NAFLD according to Brunt's criteria; however, only 70% of patients (16 out of 23) enrolled successfully completed the one year program [11]. Given the intimate relationship between insulin resistance and NAFLD, studies have also examined hepatic insulin resistance in the face of lifestyle changes. After six weeks of maintaining a low calorie diet, 33 obese subjects experienced a 32% increase in whole-body insulin sensitivity and 60% decrease in hepatic insulin resistance on average [12]. The patients also experienced an 11% reduction in liver volume, 60% decrease in liver triglyceride content as determined by magnetic resonance studies. It has become evident in the literature that interventions which result in definite weight loss can lead to significant improvements in NAFLD. Nowhere is this more evident than in the bariatric surgery population. Several studies have shown that bariatric surgery results in marked improvement of liver histopathological features such as steatosis, inflammation, and hepatocyte ballooning as well as serum liver enzymes and measurements of insulin resistance in patients with severe obesity and NAFLD [13,14].

**Table 1:** Clinical studies evaluating outcome of lifestyle interventions for obese adults and children with NAFLD

Author	Patients (n)	Study type	Therapy	Duration	Outcome
Franzese *et al*. [38]	58	Case series	Diet and exercise	Six months	85% of patients with NAFLD (19/33) with normalization or improvement in US findings of NAFLD

Huang *et al*. [11]	23	Pilot study	Diet	one year	NASH improved In 60% of patients

Johnson, NA [10]	19	Randomized, controlled trial	Exercise	four weeks	Reduction of hepatic triglyceride concentration, visceral adipose tissue volume and hepatic free fatty acids

Nobili V *et al*. [16]	53	Randomized, controlled trial	Diet and exercise	24 months	Children experienced weight loss and improved liver histology after lifestyle intervention

Oza *et al*. [39]	22	Pilot study	Diet	six months	BMI and steatosis reduced in 86% of patients who completed the study (only 32% of patients completed the study)

Palmer *et al*. [40]	39	Case series	Diet and exercise	2 to 111 months	Liver enzymes improved in patients with weight loss

Promrat *et al*. [37]	31	Randomized, controlled trial	Diet and exercise	48 weeks	More patients with lifestyle intervention had reduction in NAS score in comparison with control group

St. George *et al*. [9]	152	Randomized trial	Diet and exercise	three months	Reduction of liver enzymes in both the low-intensity and moderate-intensity groups

Ueno *et al*. [8]	25	Non-randomized, controlled trial	Diet and exercise	three months	Steatosis improved in the intervention group

Viljanen *et al*. [12]	34	Case Series	Diet	six weeks	Decreased liver volume and liver fat content, improved hepatic insulin resistance

NAFLD is also being identified more frequently in the pediatric population. These children also tend to be overweight, with a BMI above the 85% for age, or obese with a BMI above the 95% for age. Many parents of young patients with NAFLD prefer to avoid medications in favor of lifestyle modifications. Parents and physicians alike are concerned about the endpoint of medication use and potential long-term side effects of medications when started at such a young age. Thus, many parents and providers seek out programs that address lifestyle modification for obese children with metabolic complications of obesity including NAFLD. Currently available, non-invasive diagnostic tests are unable to reliably predict which patients will progress from hepatic steatosis to advanced liver disease and fibrosis; therefore, it has been suggested that diet and exercise should be the first-line of defense for preventing progression to worsening states of liver damage [15]. Evidence for this has come from a study of 53 children with NAFLD undergoing lifestyle modifications including behavioral, dietary and physical activity recommendations. It was reported that these children experienced significant improvement in BMI, NAFLD Activity Score (NAS), hepatic lobular inflammation, hepatocyte ballooning and transaminases during the 24-month study period [16]. Additionally, the patients also showed improvement in cholesterol, triglycerides and HOMA-IR (homeostasis model assessment-insulin resistance), a sensitive indicator of insulin resistance.

Each of the aforementioned studies did document improved hepatic steatosis and/or improvement in liver enzymes after lifestyle modification, but multiple clinical challenges still remain. More research is needed to determine the optimal length of time required for these types of interventions. In addition, compliance with these types of programs can be an issue for patients. Proven methods to maintain patient interest, attendance and adherence are needed as well.

## Chronic hepatitis C virus (HCV) infection

Hepatitis C virus (HCV) infection continues to be an important global health problem. It is estimated that approximately 170 million people worldwide are infected with HCV [17]. While a number of host and viral related factors are well established predictors of response to anti-viral therapy and clinical outcome, obesity and the associated metabolic complications have been increasingly recognized as independent risk factors for diminished response to therapy and more severe liver disease. Indeed, an elevated body mass index is associated with increased steatosis in patients with chronic HCV. This association appears to be present irrespective of HCV genotype [18]. In turn, increasing severity of hepatic steatosis has been associated with disease progression, worsening liver damage and fibrosis in patients with chronic HCV [19]. Although the exact mechanisms for disease progression in patients with steatosis have not been completely elucidated, it has been shown that obese patients with chronic HCV have higher circulating insulin levels, which are associated with higher levels of fibrosis [18]. A recent study of 228 patients with chronic HCV infection reported that 26% of the patients fulfilled criteria for metabolic syndrome. The diagnosis of metabolic syndrome was significantly associated with both genotype 1 HCV as well as steatosis of the liver [20]. Additionally, in a study evaluating more than 900 patients with advanced chronic HCV resistance to standard therapy, approximately 25% had both diabetes and elevated serum triglycerides. Several additional studies have also assessed the prevalence of Type 2 diabetes mellitus in patients with chronic hepatitis C infection, affecting between 8 and 25% of these patients [21,22]. Insulin resistance, as determined by HOMA-IR, and histological features of fatty liver, including degree of steatosis and presence of Mallory bodies, was associated with progressive liver disease in this cohort of patients [23]. There is evidence that the development of insulin resistance might be linked directly to the hepatitis C virus itself. Romero-Gomez *et al*. found that in 50 patients with chronic hepatitis C and a diagnosis of insulin resistance before anti-viral treatment, the patients with a sustained viral response also experienced a decrease in HOMA-IR six months after therapy was initiated in comparison to patients who were non-responders [24].

In addition to the association with progression of liver disease, there have also been studies investigating alterations in response to standard medical therapy for patients with chronic hepatitis C, obesity and hepatic steatosis. It has become clear that increased body weight has a detrimental effect on the sustained virological response to antiviral therapy in chronic HCV [25]. In fact, Hanouneh *et al. *reported that patients with chronic hepatitis C infection as well as metabolic syndrome were 3.8 times more likely to fail standard anti-viral treatment with pegylated interferon (alpha-2b or alpha-2a) and ribavirin than patients without metabolic syndrome [20]. It has also been shown that in patients with chronic hepatitis C, insulin resistance (as indicated by increased HOMA-IR) is an independent predictor of decreased viral response to standard therapy [24].

A logical approach to addressing patients with metabolic syndrome and chronic hepatitis C would be to treat the underlying liver disease and relevant components of metabolic syndrome simultaneously. Examining lifestyle interventions as a means for treating metabolic syndrome in these patients, a study of 31 obese patients with chronic hepatitis C, steatosis and overweight status who underwent 15 months of physical activity and dietary intervention showed that 68% of the patients maintained weight loss. A reduction of both serum alanine transaminase (ALT) and fasting insulin level correlated with the amount of weight lost [26]. Similarly, Hickman *et al*. reported weight loss was associated with a decrease in serum liver enzymes, hepatic steatosis and fibrosis score in obese patient with chronic hepatitis C [27]. There are no studies as yet available assessing whether these lifestyle interventions impact on response to antiviral treatment.

Two recent studies have evaluated the effect of adding an insulin sensitizer to the standard antiviral therapy with mixed results. Overbeck and colleagues studied the effects of pioglitazone in patients with hepatitis C who previously failed standard therapy with ribavirin and pegylated-interferon. However, none of the first five patients included in the trial achieved a satisfactory virological response after 12 weeks of retreatment, despite the fact that in at least three of them the insulin resistance score improved. As a result, the study was terminated early [28]. Others have suggested that this combination might be beneficial in naïve patients with chronic hepatitis C and insulin resistance. In a subsequent study, Romero-Gomez *et al. *evaluated the efficacy of metformin in naïve, genotype 1 chronic hepatitis C and insulin resistance patients in a multi-centered, randomized, double-blinded, placebo-controlled trial. Adding metformin to peginterferon and ribavirin was safe and improved insulin sensitivity. Although the study failed to show a statistically significant difference between arms, it did show an improved sustained virological response (SVR) in females [29].

Thus, larger, more definitive studies are still needed to determine the potential utility of these interventions for treatment of patients with chronic hepatitis C, insulin resistance, and obesity.

At this point only lifestyle interventions can be recommended to improve metabolic syndrome and obesity associated with chronic hepatitis C, but their effect on treatment response and long term outcome requires further study.

## Liver transplantation

Despite the various etiologies for end-stage liver disease and subsequent liver transplantation, obesity has been associated with post-transplant complications regardless of the indication for transplant [30]. Post-transplant adherence for liver recipients involves taking medications on a regular basis, keeping appointments, and making and sustaining recommended lifestyle changes related to smoking, drinking, drug use, and other high-risk behaviors. With the majority of liver transplant recipients surviving beyond the first year post-transplant, the development of metabolic abnormalities contributes significantly to morbidity and mortality in these patients [31]. Several epidemiological studies have shown that liver transplantation is associated with an increased prevalence of risk factors for cardiovascular disease, including hypertension, diabetes mellitus, dyslipidemia, and obesity, with reported rates of post-transplant metabolic syndrome ranging from 43 to 58% [32-34]. The etiology of these conditions is most likely multi-factorial including changes in body habitus as well as side effects of medications. In addition, diabetes has been shown to be an independent risk factor for mortality following liver transplantation [35]. This translates to an elevated cardiovascular event rate in post-liver transplant patients with metabolic syndrome. In a recent study it was reported that after an average of 58 months of follow-up in 118 post liver transplant patients, 30% of patients with concomitant metabolic syndrome experienced major cardiovascular events as compared with 8% of patients without metabolic syndrome [32].

Thus, it is apparent that counseling regarding management of obesity should be initiated for both patients undergoing evaluation for liver transplant and liver transplant recipients. Studies examining the long term effects of lifestyle interventions for obesity and metabolic syndrome in patients after liver transplantation have yet to be published.

## Lifestyle interventions

One of the first steps for patients with obesity and chronic liver disease is a therapeutic lifestyle change. We know that obesity management not only contributes to weight loss, but also enhances insulin sensitivity, modifies the serum lipid profiles and contributes to improved quality-of-life [9]. A recent study detailed the differences in diet between lean, overweight and obese subjects with NAFLD and hepatitis C [36]. This study showed that patients in the higher BMI groups (that is, BMI >25) consumed more high fat sources of protein, foods with a high sodium content and higher fat milk products. Another recent study has also shown the positive health effects of practical lifestyle intervention based on behavior-change theory after three months on liver enzymes and other metabolic risk factors [9]. This study by St. George *et al*. was the first on the effects of a comprehensive lifestyle intervention as opposed to intense weight loss alone in patients with liver disease. Using a large sample size and a no-treatment control group, the authors demonstrated that even small changes in weight and even weight maintenance in conjunction with improved dietary and physical activity can bring about improvements in metabolic and liver test profiles of these patients. This provides support for practitioners to encourage patients with liver disease to undertake and maintain positive lifestyle changes irrespective of whether large weight loss occurs.

We and others have demonstrated in a research setting that sustained weight loss of even a few kilograms has a beneficial effect on the biochemical and histological features of disease in overweight patients with NAFLD and chronic HCV [8,16]. In one of the only randomized, controlled trials examining the effects of weight loss on NAFLD after intensive lifestyle modification, it was reported that after 48 weeks of intervention, patients who received intensive lifestyle modification lost 9.3% of their body weight as compared to 0.2% loss in control subjects [37]. Importantly, in addition to weight loss, patients experienced improvement in the NAS activity score as well as reduction in alanine transaminase levels. These studies reflect the need for effective methods of weight reduction in an outpatient setting to become a more important aspect of clinical care. Despite the recognized benefits, achieving even modest amounts of weight loss and maintaining it in clinical practice is difficult. Intensive interventions that mimic those established by research projects are perceived as expensive and demanding on the dietetic service. Typically, patients are unable to lose or even maintain their weight, so they lose motivation. In these cases, the patients do not follow through with lifestyle interventions and/or use pharmacological interventions instead. Therefore, when discussing physical activity with patients, the discussion should focus on impediments to increasing physical activity and finding ways for patients to incorporate exercise into their lives on a regular basis. It is often helpful to separate the benefits of exercise from weight loss. Since exercise has its own benefits in terms of a sense of well-being, and improved metabolic status including insulin sensitivity and hepatic steatosis. Being discouraged about lack of weight loss during these lifestyle interventions should not be a reason to quit.

Successful introduction of lifestyle interventions into clinical practice requires facilitation and support from all levels of staff within the liver clinic. Aspects of the service structure that we believe are important to the success of the program are an initial intensive period of weekly review for 12 weeks followed by long-term support. Components of a successful program also include the commitment of a dietitian who has an interest in weight management, a consulting room physically located within the hepatology department, and support from medical and nursing staff. For patients who are unable to demonstrate long term weight loss and/or have continued liver damage, specific combinations of drugs and diet therapies may be tailored to target one or multiple metabolic pathways to regulate lipid metabolism and ultimately influence circulating plasma biomarkers of cardiovascular disease. Furthermore, combined drug/diet approaches in the change of lifestyle may reduce the number of drug prescriptions, the progressive increase in ''optimal'' drug dosage, and costs associated with pharmaceutical disease management.

## Conclusions

Individuals with chronic liver disease have lower QOL than the general population, with and without associated chronic illness. A decline in physical health is particularly apparent. When these conditions are present in patients who are also overweight or obese, QOL declines even further, along with an increased risk of other potential medical and hepatic complications. As we have discussed in detail in this review, lifestyle interventions including healthy eating, exercise and controlled weight loss have been shown to improve liver damage related to hepatic steatosis and other complications of obesity and the metabolic syndrome. Lifestyle changes should be an important adjuvant to medical therapies for patients with HCV infection, liver transplant status, and NAFLD as well as other chronic hepatic conditions. Interventions related to diet and exercises are also our best first-line therapy for patients with hepatic steatosis related to underlying obesity. Future research should focus on identifying modifiable factors that affect QOL and can be targeted for improvement. The independent inverse relationship between chronic liver disease and physical health merits further evaluation. The association between these entities warrants additional attention as these patients will increasingly be co-managed by internists, endocrinologists, and hepatologists.

## Abbreviations

ALT: alanine transaminase; BMI: body mass index; HCV: hepatitis C virus; HOMA-IR: homeostasis model assessment-insulin resistance; MS: metabolic syndrome; NAFLD: nonalcoholic fatty liver disease; NAS: NAFLD Activity Score; NASH: nonalcoholic steatohepatits; QOL: quality of life.

## Competing interests

The authors declare that they have no competing interests.

## Authors' contributions

All of the authors contributed equally to the development and writing of the manuscript.

## Pre-publication history

The pre-publication history for this paper can be accessed here:

http://www.biomedcentral.com/1741-7015/9/70/prepub
